# Measurable Indicators and Public Health

**DOI:** 10.3201/eid1009.AC1009

**Published:** 2004-09

**Authors:** Polyxeni Potter

**Affiliations:** * Centers for Disease Control and Prevention, Atlanta, Georgia, USA

**Keywords:** Art and science, emerging infectious diseases

**Figure:**
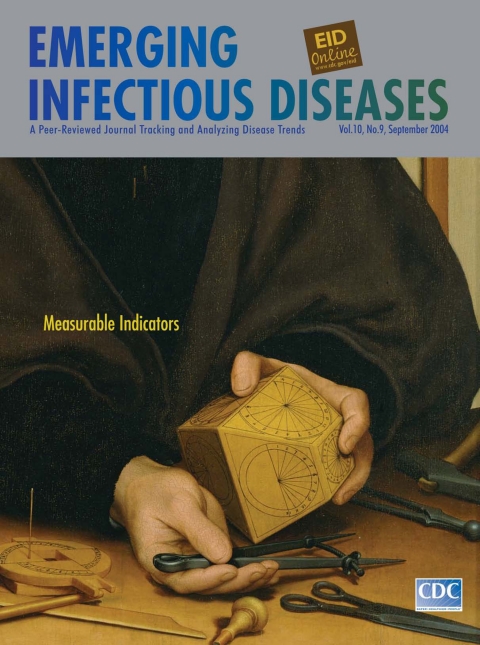
**Hans Holbein the Younger (1497–1543). Nicholas Kratzer (Detail), 1528.** Oil on wood, 83 cm x 67 cm, Louvre, Paris, France. Photo: Erich Lessing/Art Resource, New York

At its deepest level, reality is mathematical.

–Pythagoras

Art came easily to Hans Holbein the Younger. Son and student of master painter Hans Holbein the Elder, he showed extraordinary talent at a young age in his native Augsburg, a bustling commercial town in southern Germany. Fascination with Italian renaissance took him to Lombardy, where he studied the work of Leonardo da Vinci and the portraits of Lorenzo Lotto. While still a teen, Holbein moved to Switzerland and became established in Basel, where he met Erasmus of Rotterdam and other humanists under whose generous patronage he began an illustrious career as portrait and religious painter (1).

"The arts here are freezing," wrote Erasmus to Thomas More and other friends in England, urging them to support Holbein when he moved to London from Switzerland amidst the turmoil of the Reformation (2). Events of the day, among them Britain's break with the Catholic Church and the dissolution of monasteries, altered the art scene. While patronage and demand for images of religious content declined, Holbein flourished as court painter for King Henry VIII, producing woodcuts, glass and other decorative designs, and timeless portraits of the intellectual aristocracy, until his death of the plague in 1543 (3).

Part of the brief but brilliant movement known as Renaissance in the North, which included Albert Dürer and Mathias Grünewald, Holbein was able to grasp and depict the human image in a way that eluded his contemporaries (4). His portrait of Erasmus captured the essence of the famous author whose uncharismatic physique had frustrated other artists. And his single surviving portrait of Henry VIII created an enduring perception of the notorious monarch for posterity.

Holbein's celebrated portraits recorded more than the physical appearance of luminaries of his age. Many, including the portrait of Nicholas Kratzer, on this month's cover of Emerging Infectious Diseases, place the sitter in a topical context, providing valuable character clues and social commentary. Kratzer, mathematician and astronomer to Henry VIII and friend to Holbein, was a prominent maker of sundials and clocks (5). These popular objects represented practical application of mathematics and symbolized scientific knowledge, a notion wildly appreciated long before it was fully understood.

In Holbein's painting, Kratzer is preoccupied. His trancelike expression reflects detachment and lapse into some unknown calculation, an abstract reality whose nature is alluded to by the instruments at the periphery of the portrait. His smooth hands seem skilled and confident around the elaborately drawn geometric figures and the mathematical tools strewn provocatively in the foreground.

Holbein was a deliberate observer. He sorted the evidence of physical reality that he so fastidiously gathered for internal character clues. In his portraits, the stubble on the chin or smudge on the thumb was intentional, and the painstaking collection of minute and precise detail built a composite larger than its parts. This intricate composite, much often missed by the casual eye, was purposeful and focused. Free of extraneous or distracting elements, it dispassionately laid out for the viewer a meticulous image to probe for inner meaning and final interpretation. Selectively descriptive, proportional, fully cognizant of order and balance, his portraits offered a glimpse into a person's soul and an unadulterated version of the artist's perception of reality.

Domain of the artist, observation is equally domain of the scientist. Fueled by the desire to know, it drives systematic collection of data, the facts needed to formulate a unified concept of nature and the laws that govern it (6). Scientific observation, like Holbein's artistic equivalent, goes beyond the chaotic collection of facts. Sufficiently ascertained and methodically arranged and analyzed, facts form mathematical models, create measurable indicators, predict impact, and calculate costs to produce meaningful and applicable public health models. When graced with clarity of expression, like Holbein's portraits of distinguished humanists or John Snow's geospacial maps of cholera cases, observation produces good art and good science.
